# How *Microbiology* started

**DOI:** 10.1099/mic.0.001139

**Published:** 2022-02-21

**Authors:** Peter Collins

**Affiliations:** ^1^​ The Royal Society, 6–9 Carlton House Terrace, St. James’s, London, UK

## First, launch the Society

From the outset, the founders of the Society for General Microbiology (as it was called until 2015) intended to launch a journal. The journal was a key resource for promoting their vision of microbiology as a coherent and wide-ranging scientific discipline. They would soon come to realise that it was also key to financing the new Society and to carrying its message to colleagues worldwide.

First they had to get the Society itself up and running. Many of the founders had worked directly or indirectly on the second congress of the International Society for Microbiology, held in London in 1936 – the bacteriologist John Ledingham, for example, had been President of the congress and Ralph St John-Brooks had been Secretary-General. Others were clustered around the Lister Institute for Preventive Medicine in Elstree, other medical settings, various commercial laboratories, or the Society of Agricultural Bacteriologists (SAB). That 1936 congress was, in Sid Elsdon’s assessment^1^, the first time that British microbiologists of all persuasions had come together and worked hard for common purpose. The idea of microbiology as a scientific discipline distinct from its diverse fields of application began to take root in the UK at that time.

The outbreak of war naturally disrupted progress. The next occasion when ideas about the future development of the discipline could be discussed with a relatively large number of those involved was not until September 1943, at the annual conference of the SAB. The SAB was worried about its long-term future. One option appeared to be turning itself into the learned society for this wider vision of microbiology. However, influential figures such as Ledingham and St John-Brooks felt that the SAB, with its roots firmly in the world of dairying, would be too narrow a starting point, and that a wholly new body would be more likely to deliver the wider vision. Marjory Stephenson, at her ‘forceful best’ in Sid Elsdon’s recollection, persuasively argued the case for building a new society around microbiology as a whole rather than around bacteriology, so that it could include ‘parasitologists and those studying yeasts, fungi and protozoa’ [1]. She, Ledingham and St John-Brooks carried the day.

The upshot of that AGM was that Leslie Allen, newly elected President of the SAB, was deputed by the SAB to explore the possibilities of forming a new society with St John-Brooks (his near neighbour in Bushey, Hertfordshire). Together they set about the delicate process of building consensus. As a first step, they convened a meeting on 16 November 1943^2^ with representatives of the Association of Applied Biologists, the Biochemical Society, the British Mycological Society, the Pathological Society and the SAB, and with individuals working in virology, animal parasitology, dairying, type cultures and various industrial settings^3^. The meeting was chaired by John Ledingham, with Allen and St John-Brooks acting as Joint Secretaries^4^. It was agreed that there should indeed be a new society, and a new journal, its name and scope mirroring those of the society itself. This would be one of the new society’s core activities, along with holding scientific meetings, enabling microbiologists of different backgrounds to interact, and seeking to influence the teaching of microbiology. The meeting also agreed to Marjory Stephenson’s suggested name, the ‘Society for General Microbiology’.

The subcommittee^5^ appointed to take it all forward held its first meeting a month later, chaired on that occasion by Marjory Stephenson^6^. The main focus then was on how to recruit members. That involved clarifying just what was covered by the term ‘general microbiology’. Muriel Robertson thought it should include ‘the biology of diatoms, protozoa, and microscopic algae’. John Ledingham opted for ‘the bionomics of bacteria, viruses, micro-fungi, protozoa, and microscopic algae’. Arthur Stableforth went for ‘the more fundamental aspects of … taxonomy, systematics, dissociation, physiology, nutrition and ecology’. It was left to Leslie Allen and Ralph St John-Brooks to sort it out, in consultation with BCJG (‘Gabe’) Knight who had been brought into the original discussions at the suggestion of Marjory Stephenson. Their task was encapsulated in the agreed, though never used, subtitle for the society: ‘The Society for the Establishment and Extension of Common Ground between all forms of Microbiology’.

The subcommittee was clear that the new Society, and its journal, should focus on the fundamental aspects of microbiology. As a concession, it accepted the case for including papers in applied areas where there was no suitable English journal, provided always that they contained results of ‘general scientific interest’.

Leslie Allen reported these developments to the SAB in January 1944. He explained that extensive consultations had confirmed support for a society dealing with the more fundamental aspects of microbiology. He defined these aspects as ‘the biology of bacteria, viruses, micro-fungi, protozoa, and microscopic algae’, with the study of these entities to include ‘taxonomy, systematics, dissociation, variation, physiology (including oxidation-reduction potentials, vitamins, etc.), and the study of germicides, nutrition, and ecology’. The proposed society would not challenge the SAB or similar societies within their own existing spheres. The SAB welcomed these moves and, in a parallel initiative, broadened its own scope, and its name, from Agricultural Bacteriologists to the more wide-ranging Society for Applied Bacteriology, an approximate analogue in the applied sphere of the Society for General Microbiology [2].

The subcommittee, then led by Ashley Miles, set about the business of establishing formal contacts with existing learned societies that might be affected by its plans, and developing a list of potential original members, to an initial total of about 250. It also had the fun of drafting formal rules for the Society. All these were debated, tweaked and approved by the full committee^7^ on 20 March 1944. The letters to potential members included a commitment that ‘as soon as circumstances permit’, the new Society would launch a journal for papers within its disciplinary scope. Importantly, the journal would accept any suitable papers, including those that had not first been read at its meetings.

The full committee met again on 25 May 1944 to review progress and to agree a slate of initial office-holders; but it did not at that stage address the matter of who would edit the journal. Given his leadership role among British microbiologists, John Ledingham would have been the obvious choice for the inaugural presidency, but had already effectively ruled himself out. Allen and St John-Brooks therefore wrote to David Keilin, who declined through pressure of work; then to Marjory Stephenson, who declined because she felt she was not the most suitable person, though she did later accept a subsequent invitation; and finally to Alexander Fleming, who accepted. The inaugural meeting, originally scheduled for 29 September 1944, was postponed till 16 February 1945 to allow time to get all the arrangements in place. The Second World War, after all, was still in full swing, London was subject to frequent heavy air-raids and flying bombs, and ‘the difficulties of travelling resulting from evacuation and the movement of troops’^8^ were considerable.

The formal rules approved at the inaugural meeting in February 1945 included a commitment ‘as soon as practicable to issue a journal known as *The Journal of General Microbiology*’; but the post of Editor still remained unfilled. A major problem for the intended journal was wartime restrictions on the availability of paper, which effectively put stop to such new initiatives^9^. The annual subscription was set initially at one guinea (£1.05), pending an increase to cover the costs of the journal when known; all members would be entitled to receive copies of all the Society’s publications.

Muriel Robertson told Allen and St John-Brooks that, in guiding the embryonic Society to its starting point they ‘had done a noble and arduous piece of work, and we should all be grateful to you’. ‘Praise from her is praise indeed!’ wrote St John-Brooks to Allen (St John-Brooks to Allen, personal communication 20 November 1944). Their reward at the inaugural meeting was to be voted in as Joint Secretaries, so that the arduous work could continue.

**Allen and St John-Brooks [3, 4]**

Leslie Allen (1903–64) and the older Ralph St John-Brooks (1884–1963) became good friends through their shared experience in getting the Society off the ground. The fact that they happened to live near each other during the key period was a big help. It was natural that they should be appointed as the first Joint Secretaries of the new Society when it was formally launched in February 1945.

In July 1946, St John-Brooks stepped back on medical advice from his role as Joint Secretary (he had suffered from pulmonary tuberculosis 20 years previously, and had never fully recovered), to be succeeded by Kits van Heyningen. In November 1946 he was made the Society’s first Honorary Member, which came as ‘a tremendous but very agreeable surprise’. Towards the end of that year he also retired from his long-standing role as Curator of the National Collection of Type Cultures at the Lister Institute. He then had stints at the American Type Culture Collection in Washington and the equivalent Swiss body at Lausanne.

Allen wrote warmly to him in 1946 recalling fondly ‘the long fireside chats we used to have in the evenings when the project was taking shape’. ‘I feel we did a great work together’, he added. ‘The success of the new Society shows how much can be accomplished quietly by a few men of good will who work for a common aim, and who do not have to protect their personalities from the public gaze by putting on cloaks of prestige, or aggressiveness, or shyness, which make people misunderstand them. One wonders how much could be accomplished in a similar way in international affairs.’ Indeed!

Allen, then Chief Microbiologist at the DSIR Water Pollution Research Laboratory, retired from his role as Joint Secretary of the Society in March 1948, citing pressure of other commitments, to be succeeded by JG Davis.

St John-Brooks enjoyed sending Allen food parcels from America in 1947, which arrived safely, to their mutual relief. Allen would eventually write St John-Brooks’ obituary for the *Journal of General Microbiology* (JGM), but himself died before it was published; his own obituary was written by the Society’s inaugural Treasurer, Bill Bunker.

## Then, launch the journal

The Society’s newly elected Council^10^, at its first meeting on 16 March 1945, was keen to push ahead with the journal rather than dawdle until paper rationing was lifted. So it appointed a subcommittee comprising Gabe Knight, Alexander Mattick, Muriel Robertson and the two Secretaries (Allen and St John Brooks) to get things moving. By September 1945, opinion in the trade was that it should be possible to get permission from the Ministry of Production to launch a new scientific journal. By December, Council decided, on the advice of Cambridge University Press (CUP), that an annual subscription of 35/- (£1.75) would cover the additional costs of the journal with a sufficient margin for error. In February 1946, Council appointed CUP as publishers of the journal.

In February 1946, Council also, finally, addressed the issue of the Editor. It decided, pragmatically if a bit confusingly, to have two Joint Editors ‘of equal status’, one for ‘chemical’ papers and one for ‘biological’ papers^11^. It also approved the idea of a small Editorial Board, whose members (‘Associate Editors’) were to be selected primarily for their practical editorial experience and only secondarily, if at all, as covering a reasonable breadth of disciplines. This was in keeping with the Society’s aspiration of getting away from the prevailing disciplinary tribalism within microbiology. The Associate Editors would act as referees alongside the Editors (with help in peer reviewing from additional external experts as needed), and would prepare accepted papers for the press – though in practice most of that burden seems to have fallen on the two principal Editors.

Council elected Gabe Knight ahead of Marjory Stephenson’s former research student Donald Woods^12^ as the ‘chemical’ Joint Editor, but could not decide on the ‘biological’ Joint Editor; Knight invited Ashley Miles to fill that role after the meeting. Knight and Miles together selected the initial set of Associate Editors: Geoffrey Ainsworth, William Brierley, Tom Gibson, Alexander Mattick, Kenneth Smith, Arthur Stableforth^13^ and Donald Woods^14^. Brierley, Mattick and Smith were then also members of the Society’s Council, as were Knight and Miles. Both Knight and Miles were 43 years old in 1947; the others ranged from 35 years old (Woods) to 58 years old (Brierley). The group of nine – all men – included one current (Smith) and two future (Woods, Miles) Fellows of the Royal Society.

Muriel Robertson had, in November 1943, prepared a briefing on other people’s experiences in launching new learned societies and new journals, in which she commented: ‘The Editor must be hardworking and intelligent but also have some sufficient serpentine wisdom’. Beginnings, she noted, were easy: the real challenge was dealing with loss of momentum after the initial enthusiasm had worn off. Gabe Knight himself clearly had both the serpentine wisdom and the stamina required: he served as the most senior Editor until 1970, longer than anyone in the journal’s history.

The Editors and Associate Editors functioned mostly as a loose group of individuals rather than as a Board. They held formal meetings at the Linnean Society’s comfortable premises in Burlington House in September 1946, and again in December 1949, to go over various practical matters, but their plan to meet annually after that did not last long: in 1970 John Postgate was complaining that the Board had met only once in the previous 14 years. Ainsworth stepped down from his role in 1951; Miles moved to being an Associate Editor in 1951 and served in that capacity, with five of the original Associate Editors, until 1960; Gibson stayed on till 1969; and Knight outlasted them all. From 1951 to 1970, Arthur Standfast was the second Joint Editor.

The administrative office for the journal was established initially at the National Institute for Medical Research in London, where Ashley Miles was then Deputy Director and had access to secretarial support. At that early stage, the Society of course had no paid staff, but Council agreed in December 1946 that Miles’s secretary at NIMR could be paid an honorarium of £10 for each issue of the journal. The office moved to Standfast’s Department at Elstree, and his ‘indefatigable’ secretary Linda Peerless [5], when he succeeded Miles in 1951.

A notice advertising the new journal mentioned the ambition of getting the first issue of the journal published before the end of 1946, and called for papers on both specialised and general topics within the Society’s scope^15^. The Editors urged authors to make specialised papers accessible to readers from other disciplines. Twenty-seven papers were submitted by the end of 1946, and a further thirty-nine during 1947.

In November 1946, Council had the first of very many detailed discussions about the finances of the journal. Its costs greatly outweighed all other Society expenditure, so any miscalculation would have very serious consequences. On the other hand, Council quickly discovered that its profitability would make it the dominant source of Society income, outstripping members’ subscriptions by a considerable margin. Either way, finance would loom large in all policy discussions about the journal. At this early stage, the decision was simply to accept an element of advertising in the journal to mitigate any potential losses.

The caution about finance was understandable, indeed the only responsible course of action. But the journal proved to be a financial success from the outset. With the Society’s membership already approaching 600 and CUP estimating ‘trade’ sales (e.g. to academic libraries and industrial companies with relevant interests) of 350, the print run of the first issue was originally set at 1000. Allen got that increased to 1200, and the same for the second and third issues. The three issues of volume one (1947), with a total of 39 papers, cost the Society about £500 net (costs £1300, income £800); the three issues of volume two (1948), also with 39 papers, cost under £200 net because of increased sales. The print run for volume three (1949) was pushed up to 1500 after increased paper supplies had been secured. By 1955 the print run was 2750 (121 papers in six issues), and 4000 (137 papers also in six issues) in 1959. Already by the end of 1949, the journal had secured 500 external (i.e. non-member) subscriptions [6], which meant a degree of financial security.

**Three Joint Editors of JGM**

Three individuals essentially ran JGM for its first 24 years. The longest-serving was Gabe Knight, who, with Ashley Miles, got it off the ground from 1946 onwards. Miles stepped down in 1951 and was succeeded by Arthur Standfast. Knight and Standfast both retired in 1970, and were elected Honorary Members of the Society in recognition of their long and effective service.

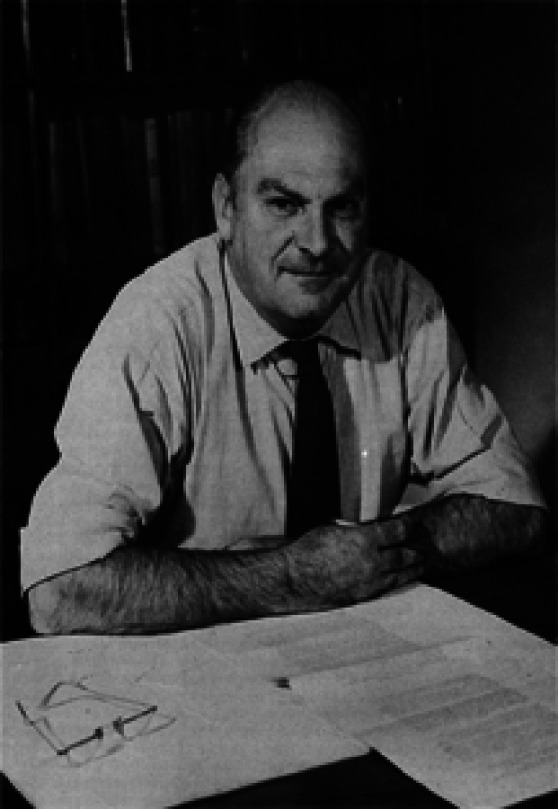

Knight’s research was mainly on bacterial growth physiology, and he published important work on the nutritional requirements of bacteria including *Staphylococcus aureus* [7]. His research career started with RK Cannan and then Paul Fildes on how the redox environment of the growth medium influences spore germination. In 1934 he moved with Fildes to a new MRC Unit on Bacterial Chemistry in the Middlesex Hospital. This was followed by four years at the Lister Institute at Elstree and, from 1943, eight years with the Wellcome Laboratories at Beckenham. In 1951 he was appointed to the first Chair in Microbiology in the first UK university Department of Microbiology, at Reading, where one of the buildings is now named after him. He retired from Reading in 1969. An avid and learned Francophile, he translated books on and by Diderot and Stendhal, and an internet search is as likely to connect him with that activity as with microbiology.

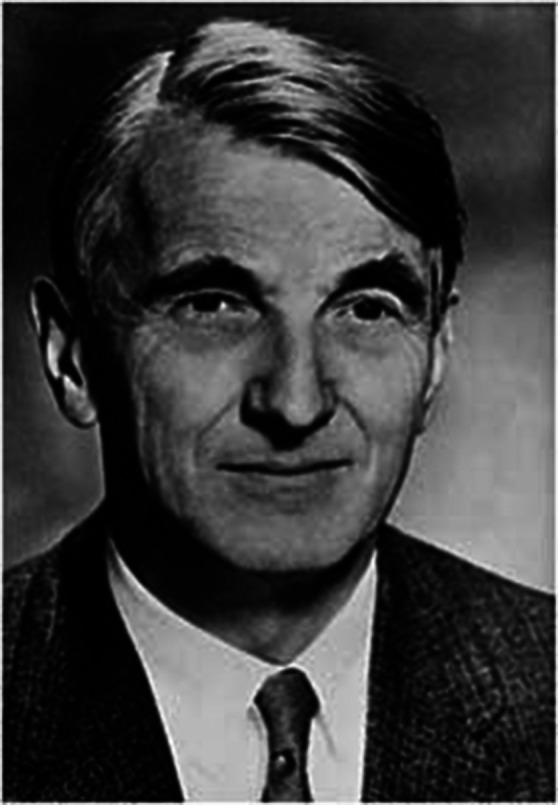

Miles, ‘essentially a medical microbiologist of the classical type’, started in medicine and then held posts in bacteriology and pathology before taking the Chair in Bacteriology at University College Hospital in 1937 [8]. This was soon combined with a range of war-related responsibilities. There followed a stint as Deputy Director at NIMR, with a particular focus on biological standards, during 1946–52, and then appointment as Director of the Lister Institute and Professor of Experimental Pathology at London. He retired from both these posts in 1971. He was elected FRS in 1961 and served as Biological Secretary of the Royal Society 1963–68. Miles’s wife Ellen, with whom he co-published several research papers, was a half-sister of Roald Dahl.

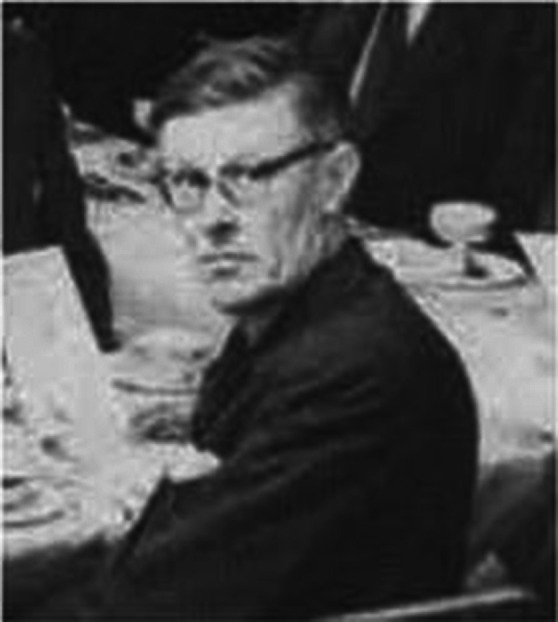

Standfast (1905–90) joined the Elstree Laboratories of the Lister Institute in 1945, working on the preparation and study of bacterial vaccines (based on annual reports of the Lister Institute. Muriel Robertson was working in protozoology at the Institute when he arrived. At first Standfast seems to have been a one-man band, but he was gradually able to build up a team and came to serve as Head of the Bacterial Vaccine Department for 26 years, until his retirement in 1972. For the whole of this period his research was mainly on pertussis, cholera, typhoid and tetanus vaccines, and he organised their commercial production at Elstree.

## *General* microbiology?

Given the Society’s origins and aspirations, it was inevitable that the disciplinary distribution of the papers published in its journal should attract close attention. Analyses presented at successive AGMs highlighted the fact that the bacteriological heritage of the Society was fading too slowly, to the occasional frustration of the Editors. Already at the September 1947 AGM, Ashley Miles remarked: ‘The Editors realise that the subjects covered so far do not represent all branches of microbiology, but hope that appropriate papers from all disciplines will be submitted to make the Journal more representative of general microbiology’. A few months later, in March 1948, Gabe Knight reiterated the message: ‘The Editors are aware that the Journal does not yet fully reflect in a balanced way all the different disciplines which the Society was formed to help unify. The remedy lies in the hands of the members’. And Ashley Miles again in April 1949: ‘The Society will notice that bacteriological papers still predominate. … The attainment of the desirable end of making the Journal more representative of general microbiology … lay in the hands of Members of the Society’.

These messages about the ‘great preponderance of bacteriological papers’ continued year after year. Finally, at the 1954 AGM, there was a slight change of tone: ‘The distribution of papers in 1953 showed an increase over 1952 in papers in Microbial Genetics, Mycology and Viruses, which the Editors feel is a good sign’. But only a slight change. By the 1956 AGM progress had stalled, and the Editors commented languidly: ‘It would seem that the Society gets the journal it writes, if not the journal it deserves’. But it was at least attracting about a third of its papers from outside the UK: ‘The journal can hardly be accused of being too insular’.

Yet the project to embed the distinct discipline of microbiology in the consciousness of the British scientific community did succeed over time. Academic appointments, university departments, undergraduate courses did gradually come to reflect that recognition. By 1994, when the journal dropped the word ‘general’ from its name and graduated to plain *Microbiology*, it was not giving up on its mission but rather signalling that the message had been accepted and no longer needed to be spelt out. But that’s another story.

## Notes

1 The sources for this paper are largely to be found within the uncatalogued archives of the Microbiology Society, held at the Wellcome Collection, and have not been itemised individually here. These archives include some correspondence, random Council papers, and valuable typescript histories of the Society including a 34-page essay by Sid Elsdon (President, 1969–72) on the foundation of the Society, and a 64-page piece by Doug Watson (Treasurer, 1979–87) on the first 50 years, based on analysis of Council minutes. I have also had access to a full set of Council minutes. Other sources are referenced below as relevant.

2 The minutes wrongly give the date as Tuesday 17 November, but it was actually Tuesday 16 November.

3 Those present on 16 November were: Leslie Allen, Christopher Andrewes, Bill Bunker, DHF Clayson, CE Coulthard, JT Duncan, Alexander Fleming, HB Hutchinson, Gabe Knight, John Ledingham, Walter Macleod, EW Mason, Alexander Mattick, Ashley Miles, Walter Morgan, Muriel Robertson, Ralph St John-Brooks, Kenneth Smith, Arthur Stableforth, Marjory Stephenson, Henry Thornton, Samuel Wiltshire and WR Wooldridge, with Sam Bedson, WJ Dowson and Paul Fildes sending apologies.

4 Ledingham subsequently took more of a back seat as he found ‘the burden of presiding over our gatherings too exacting for him’, and he died on 4 October 1944 after a brief illness [9].

5 Its members were: HJ (‘Bill’) Bunker, Gabe Knight, John Ledingham, Alexander Mattick, Ashley Miles, Muriel Robertson, Arthur Stableforth, Marjory Stephenson, Samuel Wiltshire; with Ralph St John-Brooks and Leslie Allen as Joint Secretaries [10].

6 At her own request, Marjory Stephenson remained an active participant but was spared the burden of chairing subsequent meetings, which were taken instead by Ashley Miles.

7 Essentially, the group that had met on 16 November 1943.

8 Undated circular from Allen and St John-Brooks postponing the inaugural meeting, circa October 1944.

9 By 1945, for example, newspapers were restricted to 25 % of their pre-war consumption of paper.

10 It was, somewhat unhelpfully, termed the ‘Committee’ until July 1962, but for clarity I have used ‘Council’ throughout.

11 In practice, they were always identified simply as ‘Editors’.

12 Woods instead served as an Associate Editor, from 1946 to 1960. He was later nominated to succeed David Henderson as SGM President for the 1965–7 term, but died before he could assume office. His place was taken by Percy Brian.

13 Stableforth, Senior Research Officer and later Director of the Weybridge Central Veterinary Laboratory, complained that the initial slate of six Associate Editors lacked any veterinary microbiologists, and thereupon was added to the team.

14 Woods, then Reader in Microbiology at the Oxford Department of Biochemistry, would in 1955 be appointed to the country’s first Chair in Chemical Microbiology, in the same department.

15 In fact, the first issue, labelled January 1947, appeared at the end of March 1947 – the first of many missed deadlines. Leslie Allen to SE Jacobs (original member of the SGM), 27 March 1947: ‘The first issue of the *Journal of General Microbiology* is expected to be out this week. It has been continually delayed for one reason and another, recently because of the coal shortage’.
